# Hydrogen Embrittlement Susceptibility of a Newly Developed Grain-Refined Ultra-High Strength Steel

**DOI:** 10.3390/ma18050987

**Published:** 2025-02-24

**Authors:** Wanqing Lv, Wenchao Yu, Zhifang Wu, Yongming Yan, Jie Shi, Maoqiu Wang

**Affiliations:** 1 State Key Laboratory of Advanced Refractories, Wuhan University of Science and Technology, Wuhan 430081, China; 2726064445@wust.edu.cn; 2 Central Iron & Steel Research Institute Company Limited, Beijing 100081, China; yanyongming@nercast.com (Y.Y.); shijie@nercast.com (J.S.); wangmaoqiu@nercast.com (M.W.)

**Keywords:** ultra-high-strength steel, fine grains, hydrogen embrittlement, slow strain rate test, thermal desorption spectrometry

## Abstract

The hydrogen embrittlement susceptibility of a newly developed 1700 MPa-grade ultra-high-strength steel with a primary austenite grain size of 4 μm was studied and the mechanical properties and microstructure were characterized. The results show that the hydrogen content in the steel increases with the extension of charging time: the value reached 0.35 wppm with a charging time of 96 h. On the contrary, the fracture mode of the experimental steel remained ductile after hydrogen charging, and the elongation and the section shrinkage showed little difference, indicating an excellent resistance to hydrogen embrittlement, which could be ascribed to the refined microstructure and good cleanliness of the experimental steel.

## 1. Introduction

With the improvement of social requirements for energy conservation and emission reduction, the light weight and high performance of mechanical vehicles have become an important development trend, coming with the application demand for steels with higher strength levels [1]. Currently, martensitic steels remain predominant among ultra-high-strength steels. However, when the tensile strength of martensitic steels exceeds 1200 MPa, the issue of hydrogen-induced delayed fracture gradually emerges. As the strength level of the steel increases, hydrogen embrittlement susceptibility escalates markedly [2,3,4]. The damaging effects of hydrogen in iron and steel have been recognized for over a century. When hydrogen infiltrates steels, it leads to plastic loss, resulting in decreased load-bearing capacity and the initiation of micro-cracks or early fractures, which is known as hydrogen embrittlement (HE) [5,6,7]. The occurrence of HE is influenced by various factors. Some scholars [8,9] have examined the impact of grain size on the HE susceptibility of martensitic steels. The results show that grain refinement is beneficial to reduce the HE susceptibility of the material. The fracture mode of the material may change from intergranular fracture to transgranular fracture. This shift is primarily due to an increased number of grain boundaries, which can capture more hydrogen atoms and thereby minimize hydrogen accumulation within the material. Some researches [10,11,12] have explored the effects of titanium (Ti) and molybdenum (Mo) on the HE resistance of martensitic steels. Results show that hydrogen in the material is preferentially captured by MC and M_2_C type carbides, which can act as hydrogen traps to capture free hydrogen atoms, increase the critical hydrogen content of the material, and mitigate the intergranular fracture tendency caused by hydrogen segregation at the grain boundaries, thus leading to a reduction in hydrogen embrittlement susceptibility. Depover et al. [10] investigated the hydrogen capture behavior of TiC in Fe-C-Ti martensitic steel. The results indicate that the addition of Ti reduces the HE susceptibility of the material and the hydrogen content captured by the alloy increased with the amount of TiC precipitated phase. Liu et al. [12] studied the effect of Mo element on the HE susceptibility of martensitic steel. The results indicate that steel with higher Mo content exhibits improved resistance to HE and a higher critical hydrogen concentration, which is primarily attributed to an increase in the precipitation of M_2_C carbides. In addition, the cleanliness of the steel also has a significant influence on the HE susceptibility of materials. Existing research [13,14,15] has demonstrated that impurity elements such as sulfur (S) and phosphorus (P) in steel readily interact with enriched hydrogen, weakening the grain boundaries and resulting in intergranular fractures, which exhibit typical characteristics of HE.

Currently, the demand for higher-strength steel is increasing both domestically and internationally. HB500 level ultra-high-strength steels exhibit excellent strength–toughness balance and wear resistance, contributing not only to a longer lifespan and reduced weight in industrial applications but also to cost savings [16]. Consequently, it has become a preferred material for wear-resistant applications, such as in mining machinery. However, their high strength also gives rise to higher susceptibility to HE, leading to brittle fracture when exposed to hydrogen and resulting in a decline in mechanical properties [3]. The development of high-performance steels with better HE resistance remains a hot topic. Among all the strengthening methods, grain refinement has drawn more attention due to its favorable effect on both the strength and toughness of martensitic steels. However, achieving a grain size smaller than five microns is challenging.

In this manuscript, the grain size of a HB500 level ultra-high-strength steel, 30Mn2MoTi, is refined to 4 μm by adding the microalloying element Ti associated with a sufficient amount of Mo and, controlling rolling during production [17]. The steel shows excellent mechanical properties, which has drawn more attention in the automotive and mining industries and other fields. However, since the steel is a type of special equipment that needs to be used in various extreme environments, such as in high humidity, which can lead to brittle fracture, it is necessary to investigate its susceptibility to hydrogen embrittlement for further application. Therefore, the mechanical properties and microstructure were analyzed. Factors related to its susceptibility were analyzed by thermal desorption spectrometry (TDS) and slow strain rate tensile (SSRT) tests after electrochemical hydrogen charging, The high performance of HE resistance of the experimental steel was discussed, providing data to support the dissemination and application of this experimental steel and could provide a new reference for research on HE susceptibility in ultra-high-strength metallic materials.

## 2. Experimental

The experimental steel is a newly developed low-alloy ultra-high-strength steel (30Mn2MoTi) produced by an industrial production system consisting of converter smelting + continuous casting + continuous rolling. The thickness of the steel plate is 8 mm and the chemical composition that was determined by ourselves is shown in Table 1. The hot rolled steel plate was heated at 900 °C for 30 min, followed by water cooling to room temperature, and then tempered at 180 °C for 2 h and air cooled to room temperature to obtain the experimental steel plate.

The corresponding specimens were cut directly from the steel plate for microstructure and mechanical analysis. The specimens were etched by 4% nitric acid alcohol solution after mechanical grinding and polishing, and the microstructure was observed using an optical microscope (OM, LEICA DMi8, Wetzlar, Germany, 2010) and a Quanta 650 FEG thermal field emission scanning electron microscope (SEM, Quanta 650 FEG, Houston, TX, USA, 2015). The other specimens were cut into 300 μm thin sections by wire cutting and then mechanically grinded, electrolytically polished, and observed on an FEI Tecnai G2 F20 field emission transmission electron microscope (TEM, FEI Tecnai G2 F20, Hillsboro, OR, USA, 2010). The amount and the element fractions of carbides in the steel were determined through analyzing electrolytically extracted carbide residues by X-ray diffraction using Co radiation. Tensile tests were conducted using a WE-300 hydraulic tensile testing machine (WE-300, China, 2015) in accordance with the ISO 6892-1: 2019 [18] standard, with a strain rate of 0.00025 s^−1^. Impact toughness tests were conducted using an NI 300 impact testing machine (NI 300, China, 2015) in accordance with the ISO 148-1: 2016 [19] standard at −40 °C. The size of the impact specimen was 10 × 7.5 × 55 mm.

**Figure 1:**
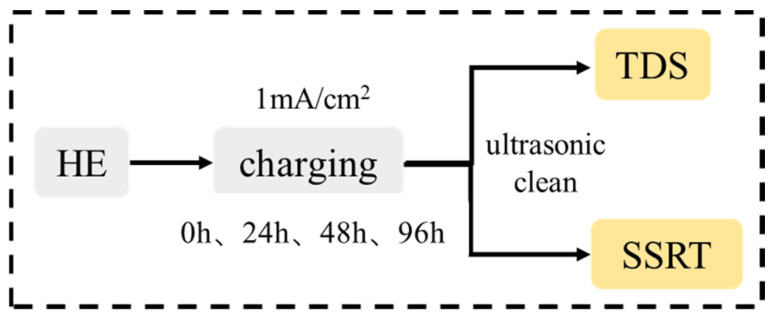
The test flowchart of TDS and SSRT.

**Figure 2:**
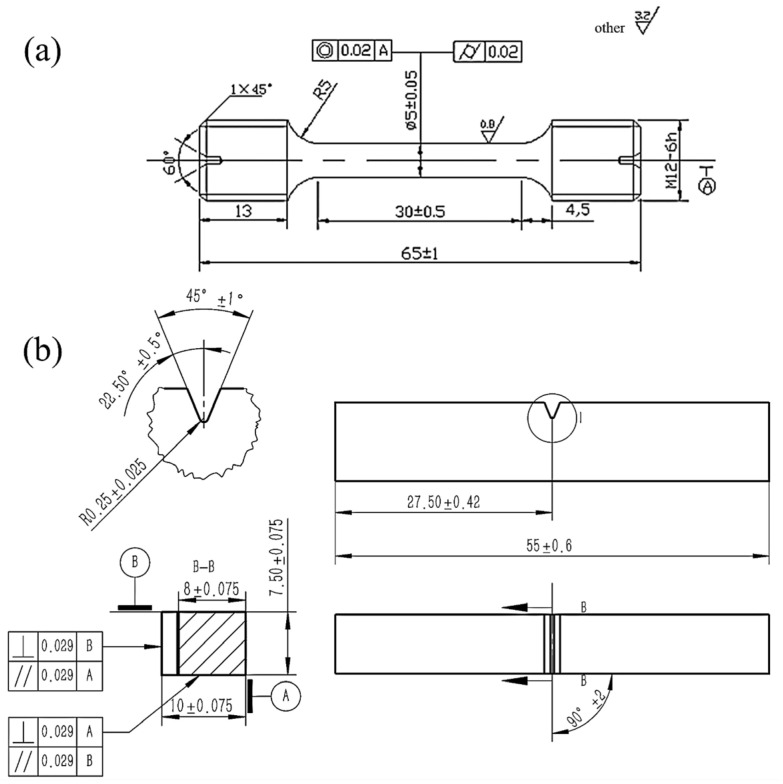
Schematic diagram of smooth tensile and impact specimens ((**a**): tensile, (**b**): impact).

The test flowchart of TDS and SSRT are shown in Figure 1. The smooth specimens (Figure 2) and the TDS specimens (∅5 mm × 25 mm) were electrochemically charged in 0.1 mol/L NaOH aqueous solution. The hydrogen charging current density was 1 mA/cm^2^ and the charging time was 0 h, 24 h, 48 h, and 96 h, respectively (0 h is the uncharged specimen). Each condition was performed for two trials and then the average of the results was calculated. After charging, the TDS specimens were ground and cleaned ultrasonically in ethanol and then directly used for TDS analysis (TDS, HTDS-002, Nagano, Japan, 2010). The desorption hydrogen content was obtained by integrating the measured desorption rate–time curve. The smooth tensile specimens after charging were subjected to slow strain rate tensile test (SSRT, WDML-300 kN, Beijing, China, 2009). The tensile speed was 0.01 mm/min, which was equivalent to a strain rate of about 1.67 × 10^−6^ s^−1^. Some scholars [20] have studied that for tempered martensitic steel, when the tensile speed is lower than 0.01 mm/min, the hydrogen in the steel matrix has uniformly diffused during the tensile process, and the tensile strength no longer decreases significantly with the decrease in strain rate. Therefore, this experiment chose a tensile speed of 0.01 mm/min. The fracture morphology of the smooth tensile specimens was observed by SEM.

## 3. Results

### 3.1. Microstructure and Mechanical Properties

**Figure 3:**
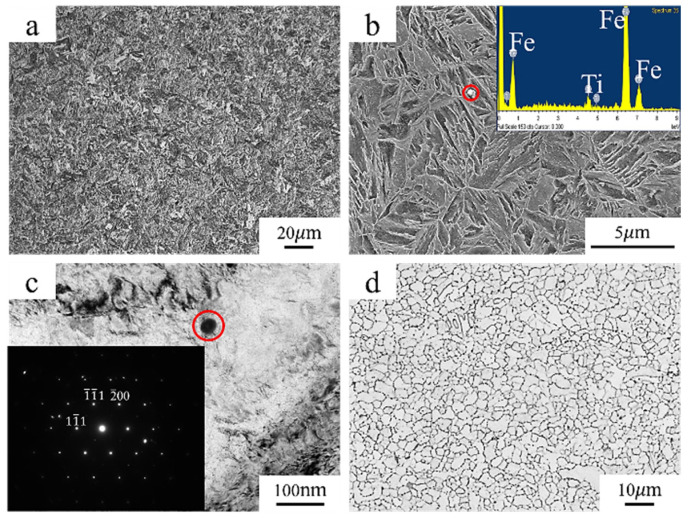
Microstructure of experimental steel: (**a**) OM; (**b**) SEM; (**c**) TEM; (**d**) grain size. The red circle in the figure marks the TiC precipitated phase.

**Figure 4:**
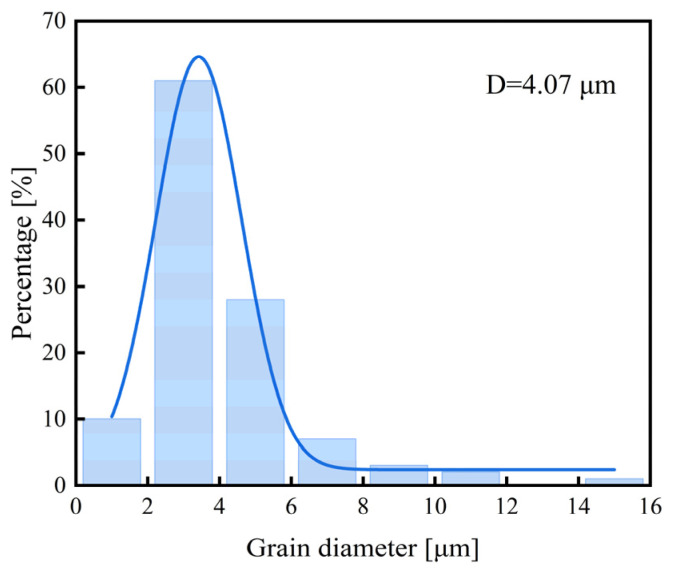
Distribution of average grain diameter.

The microstructure morphology of 30Mn2MoTi steel is illustrated in Figure 3. The results reveal that the metallographic structure of the steel predominantly consists of lath martensite. White spherical TiC precipitates can be observed in the SEM image (Figure 3b) and were confirmed by TEM analysis (Figure 3c). The primary austenite grain of 30Mn2MoTi steel is revealed, and the average size is approximately 4 μm, as shown in Figure 3d and Figure 4.

The mechanical properties of the experimental steel are shown in Table 2. The tensile strength and yield strength are 1760 MPa and 1520 MPa, respectively, leading to a high yield ratio (yield ratio = *Rp0.2*/*Rm*) of 0.86. The elongation and shrinkage are 12.5% and 44.5%, respectively, indicating excellent plasticity at such a high strength level. The impact toughness is 26 J.

### 3.2. TDS Curve and Hydrogen Content

**Figure 5:**
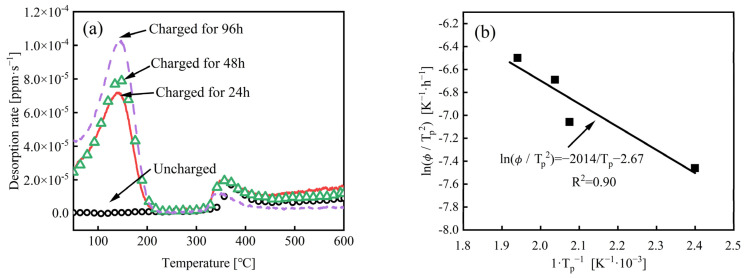
Different charging conditions of the experimental steel: (**a**) hydrogen desorption curves; (**b**) the activation energy of hydrogen desorption.

Figure 5a presents the hydrogen desorption curves of the experimental steel with different charging times (0–96 h), and the measured hydrogen contents and the corresponding peak temperatures are shown in Table 3. The heating rate was 100 °C/h with a maximum temperature of 800 °C. It is widely accepted that hydrogen desorption below 300 °C corresponds to diffusible hydrogen [21,22]. Viewing the figure, the uncharged specimen exhibits a single hydrogen desorption peak with a low intensity at 360 °C and the calculated hydrogen content is approximately 0.03 wppm. The desorption curve of the uncharged specimen remains approximately linear before 360 °C, indicating a low content of diffusible hydrogen in the steel. In contrast, the specimens filled with hydrogen display an additional desorption peak at lower temperatures around 145 °C, which corresponds to diffusible hydrogen primarily generated by electrochemical hydrogen charging. The hydrogen content at this peak varies depending on the charging conditions. From Figure 5, it can be found that the hydrogen desorption peak at 145 °C increases with the extension of the hydrogen charging time, indicating that the diffusible hydrogen content in the charged specimen is increasing, and the hydrogen content gets to a maximum of 0.35 wppm when the charging time reaches 96 h. The temperature of Peak 2 is 360 °C. The hydrogen content corresponding to Peak 2 in Table 3 shows little difference, which indicates that electrochemical hydrogen charging at room temperature does not have a significant effect on Peak 2 at high temperatures.

Different hydrogen traps can be identified based on the hydrogen desorption activation energy (*Ea*). Choo and Lee proposed a method for determining the *Ea* value using the peak temperature of hydrogen desorption [14]. The hydrogen desorption rate can be expressed as follows:

(1) $$\frac{dx}{dt}=A\;(1-X)\exp(-\frac{E_{a}}{\mathrm{RT}})$$

where *X* represents the proportion of hydrogen desorption, A is an empirical constant, R is the gas constant, and T is the absolute temperature. It can be observed that when the specimens filled with hydrogen are subjected to a constant heating rate Φ, the peak of the hydrogen desorption rate curve corresponds to a specific hydrogen trap. When the first derivative of Equation (1) equals zero, the hydrogen desorption rate at that point is maximized. By applying differentiation to both sides of Equation (1) and setting the derivative to zero, the following equation can be obtained:

(2) $$E_{a}\Phi /RT_{p}^{2}=A\;\exp(-E_{a}/RT_{p})$$

where $T_{p}$ is the peak temperature. By taking the logarithm of two sides of Equation (2) and the partial derivation of 1/$T_{p}$, the following can be obtained:

(3) $$\frac{\partial ln(\Phi /T_{p}^{2})}{\partial \left(1/T_{p}\right)}=-\frac{E_{a}}{R}$$

Therefore, by measuring the hydrogen desorption peak temperature $T_{p}$ corresponding to different constant heating rates *Φ*, the relationship curve between the hydrogen desorption peak $ln(\Phi /T_{p}^{2})$ and 1*/*$T_{p}$ can be drawn, and the hydrogen trap activation energy *Ea* can be obtained by the slope of the curve.

Figure 5b is the relationship between the hydrogen desorption peak $ln(\Phi /T_{p}^{2})$ and 1/$T_{p}$ of the experimental steel Peak 1, and the hydrogen desorption activation energy *Ea* corresponding to the peak temperature (145 °C) is calculated to be 18.6 kJ/mol.

### 3.3. Slow Strain Rate Tensile Test

**Figure 6:**
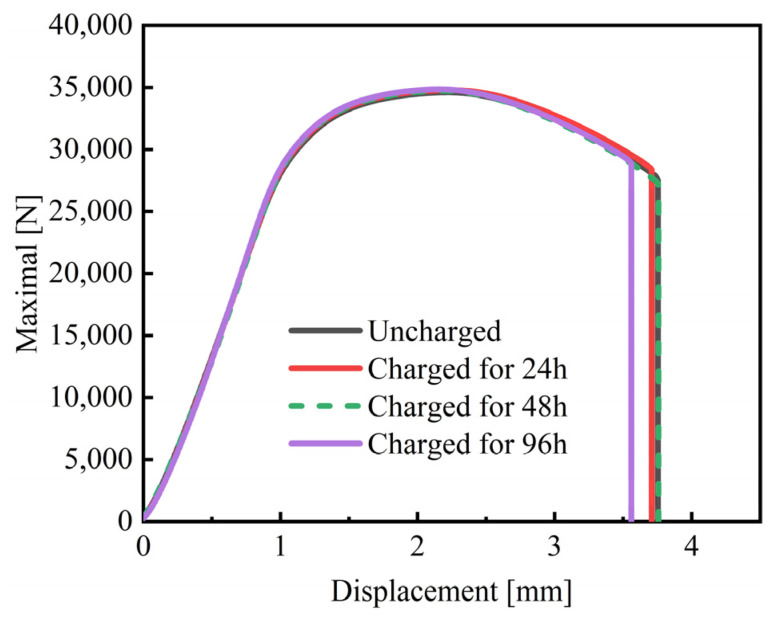
Load–displacement curves of the experimental steels.

Figure 6 presents the SSRT curves of 30Mn2MoTi steel charged by different conditions, and the corresponding mechanical properties are shown in Table 4. It can be observed that both uncharged and charged specimens show obvious yielding and necking prior to final fracture. The uncharged specimen shows similar mechanical properties as those obtained by the tensile test. With the extension in charging time, the hydrogen content in the steel increased, while on the contrary, the SSRT curves of the experimental steel remained unchanged, and the tensile strength, the elongation after fracture, and the section shrinkage showed little difference. Even for the specimen with a hydrogen content of 0.35 wppm, a tensile strength of 1768 MPa was obtained, along with a slightly smaller elongation after fracture and a section shrinkage of 42% and 11.5%, which were consistent with the uncharged specimens (Figure 6, Table 4).

**Figure 7:**
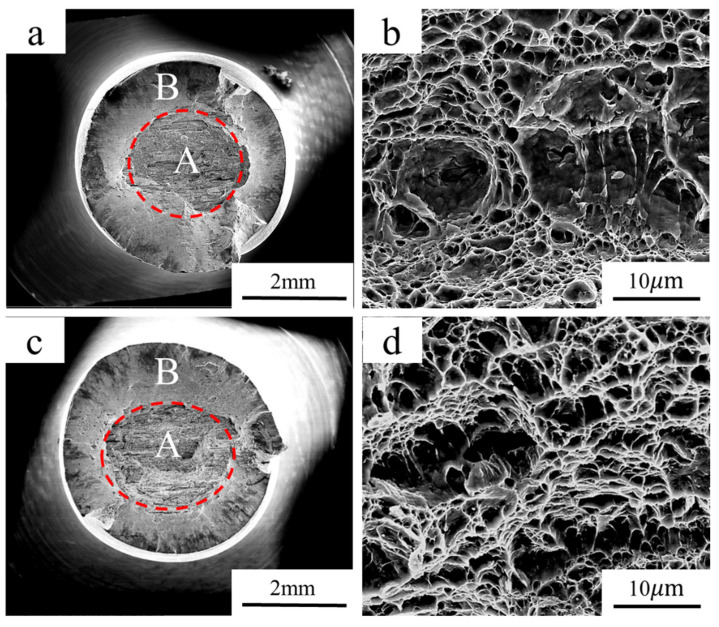
Fracture morphology of the slow strain rate tensile specimens: (**a**,**b**) uncharged; (**c**,**d**) charged for 96 h (A: central region; B: peripheral region).

Figure 7 presents the SEM morphology of the SSRT specimen fractures of the experimental steel. As illustrated in Figure 7a, the uncharged specimen displays two distinct regions, corresponding to the crack initiation and propagation in the central region and final unstable fracture in the peripheral region, respectively. The figure of the central region at a higher magnification shows that the crack initiation site consisted of dimples (Figure 7b), indicating good plasticity. The peripheral region was actually the shear lip with the width ranging from 200 to 300 μm, which was also composed of dimples. However, the dimples in this region were significantly finer than those in the crack initiation region. Thus, it can be confirmed that the steel has a typical micro-void coalescence (MVC) ductile fracture mode in the absence of diffusible hydrogen. Similarly, the specimens filled with hydrogen displayed the same morphology. The fractures also consist of two regions and the dimensions showed little difference compared to those of the uncharged specimen. Yielding and necking phenomena were observed prior to fracture (Figure 6), while the characteristics of quasi-cleavage fracture and intergranular fracture typical of HE were not observed. In this sense, the fracture mode of the charged specimens remained ductile even after charging for 96 h (Figure 7d).

## 4. Discussion

### 4.1. Hydrogen Embrittlement Susceptibility of Ultra-High-Strength Steels

**Figure 8:**
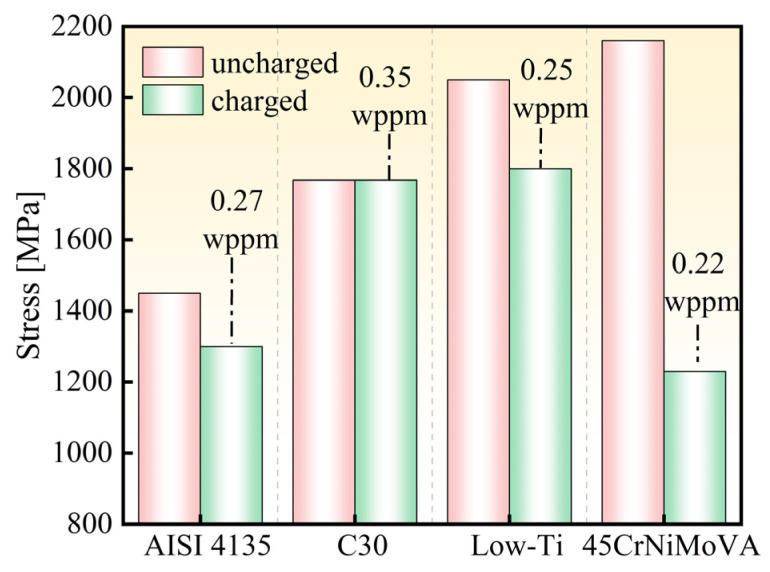
The decrease in fracture stress of different-strength steel after charging.

In recent years, the issue of HE in ultra-high-strength martensitic steels has garnered significant attention. Table 5 and Figure 8 summarize some research findings on HE susceptibility in ultra-high-strength steels with varying strength levels. For AISI 4135 steel, the tensile strength decreases from 1450 MPa to 1300 MPa when the hydrogen content reaches 0.27 wppm [2]. Low-Ti martensitic steel has a tensile strength of 2050 MPa, which drops to 1800 MPa at a hydrogen content of 0.25 wppm, exhibiting brittle fracture characteristics [23]. For 45CrNiMoVA steel, which has a tensile strength of 2160 MPa, the strength drops to 1230 MPa when the hydrogen content is 0.22 wppm [24]. In contrast, the tensile strength of 30Mn2MoTi steel is about 1768 MPa, and there is no obvious change in strength when the hydrogen content reaches 0.35 wppm. The fracture mode remained ductile with a multitude of dimples distributed on the fracture (Figure 7). This result indicates a much lower HE susceptibility of 30Mn2MoTi steel compared to reported ultra-high-strength steels.

### 4.2. Low Hydrogen Embrittlement Susceptibility of 30Mn2MoTi

Previous studies [25,26] have suggested that the HE susceptibility of ultra-high-strength steels is influenced by factors such as carbides, grain size, and cleanliness. To investigate the factors contributing to the excellent hydrogen embrittlement (HE) resistance of 30Mn2MoTi steel, the above aspects are analyzed.

Wei et al. [27] have explored the effect of titanium (Ti) on the HE resistance of alloy steels. The results show that fine TiC particles (<70 nm) can effectively capture hydrogen and reduce HE susceptibility. In this sense, the precipitated carbides in 30Mn2MoTi steel were extracted and characterized, and the results are presented in Table 6. The identified contents of carbides are M_3_C and MC. The major alloy element in M_3_C carbides is iron (Fe), though traces of other elements such as manganese (Mn) and molybdenum (Mo) are also detected. In contrast, titanium (Ti) is the main element in MC carbides with a small amount of Mo. In addition, it can be found that the Ti content in MC carbide is 0.060, which indicates that basically all Ti elements in 30Mn2MoTi steel have essentially precipitated.

**Figure 9:**
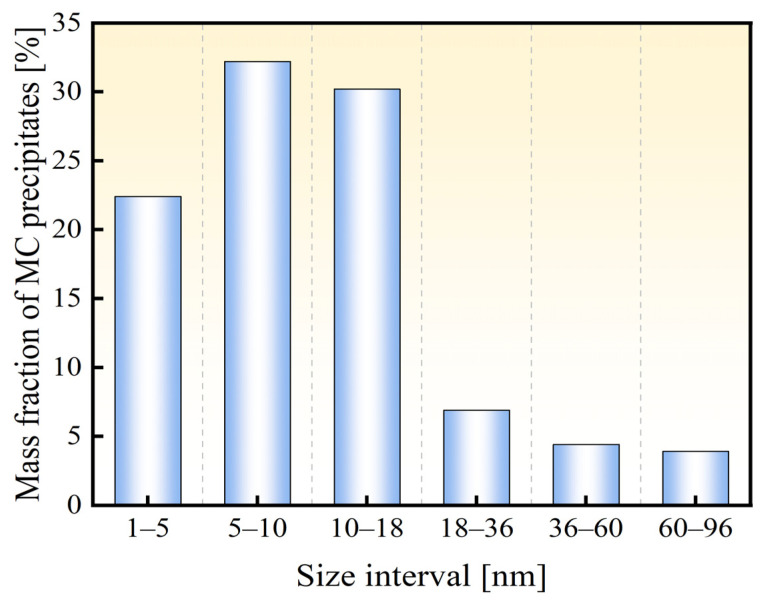
Particle size distribution of MC phase in the experimental steel.

Figure 9 shows the particle size distribution of MC-type precipitates in the experimental steel. Based on the figure, particles smaller than 70 nm account for 95% of the total amount, while, on the contrary, the sizes of M_3_C-type carbides are larger than 1 μm. Though the amount of M_3_C carbides is about 3.9 times as much as that of MC carbides, the trapping effect is mainly contributed by the latter because of their much smaller size, as reported in the literature [10]. TiC particles precipitated in the steel matrix can act as hydrogen traps to capture hydrogen during the charging process, limiting the diffusion of hydrogen and reducing the HE susceptibility of the material. However, in this manuscript, the amount of hydrogen trapped by carbides, which corresponds to the desorption peaks above 300 °C in the TDS curves, is much smaller compared to that of diffusible hydrogen. Additionally, hydrogen trapped by carbides is difficult to move and diffuse; thus, its effect on the resistance of HE susceptibility is limited.

Wang et al. [28] reported that the TDS curve of a low-carbon boron-containing steel, which was quenched at 880 °C and tempered at low temperatures, exhibited a low-temperature peak between 150 °C and 180 °C, corresponding to activation energies ($E_{a}$) of 16.1 kJ/mol and 13.8 kJ/mol, respectively. The authors considered that this peak is due to hydrogen captured at grain boundaries. Jin et al. [29,30] have examined hydrogen trapping behavior in martensitic steels and noted that the peak temperatures corresponding to hydrogen captured by grain boundaries in the TDS curves were 148 °C, 163 °C, and 159 °C, with corresponding hydrogen desorption activation energies of 16.9 kJ/mol, 15.6 kJ/mol, and 14.7 kJ/mol, respectively. Considering that the composition and microstructure of the experimental steel are similar to those above experimental steels, the Peak 1 temperature of 145 °C (Figure 5a) is relatively close, and the $E_{a}$ of the experimental steel is calculated to be 16.8 kJ/mol, so it can be inferred that the hydrogen traps corresponding to Peak 1 of the experimental steel are grain boundaries. During electrochemical charging, hydrogen in the steel is primarily absorbed by the grain boundaries, which serve as hydrogen traps.

Several studies [31,32] have examined the impact of different microstructural morphologies on HE susceptibility, and results have revealed that grain refinement effectively reduces the HE susceptibility of martensitic steel. The grain size of the experimental steel is approximately 4 μm (Figure 3d), which is much finer than that reported for AISI 4135 martensitic steel (11 μm) [2]. The fine grain of the experimental steel can be attributed to the controlled processing parameters employed during production and the addition of Ti and Mo as microalloying elements, which has been reported in our previous paper [33]. With grain refinement, the area of grain boundaries per unit volume increases, which can act as effective hydrogen traps to attract and capture significant amount of hydrogen [34]. When hydrogen contents are comparable, steels with finer grains can more effectively disperse hydrogen atoms to reduce the accumulation within the grain and thus downgrade HE susceptibility. Consequently, despite the hydrogen content being higher than that reported for AISI 4135 martensitic steel (0.27 wppm), the experimental steel maintains good strength and plasticity, as evidenced by the presence of numerous dimples in the slow tensile fractures (Figure 7d).

In addition, impurity elements in steels can interact with diffusible hydrogen concentrated at the grain boundaries, leading to weakened boundaries and resulting in intergranular fracture [12,13]. Compared to AISI 4135 martensitic steel [2], the cleanliness of the experimental steel has improved significantly, particularly with the S content reduced from 0.014% to 0.002%, representing a nearly sevenfold decrease, while the P content decreased from 0.019% to 0.009%. The low S and P contents in the experimental steel resulted in fewer impurity particles at the grain boundaries. Furthermore, the fine grains of the experimental steel could enhance the diffusion path of hydrogen within the steel, promote a more uniform distribution of hydrogen, and decrease the likelihood of hydrogen aggregation with impurity particles and the risk of brittle fracture. Shao et al. [35] found that steel exhibiting superior impact toughness displays a slower crack growth rate and enhanced resistance to hydrogen embrittlement. Grain refinement effectively contributes to the toughness improvement of the experimental steel and increases the number of grain boundaries, making crack propagation more complicated and thus increasing the resistance of crack propagation. As a consequence, 30Mn2MoTi steel demonstrates relatively low hydrogen embrittlement susceptibility compared to AISI 4135 martensitic steel, despite having a higher strength. In contrast, AISI 4135 steel, which has larger grains and higher contents of impurity elements, exhibits intergranular fracture characteristics, and the fracture mode is brittle fracture [2].

In summary, the newly developed ultra-high-strength steel, 30Mn2MoTi, exhibits refined grains due to the combination of Ti and Mo microalloying and an optimized production method, resulting in a high strength of 1700 MPa combined with exceptional plasticity. The fine microstructure and low levels of impurity elements contribute to its low susceptibility to hydrogen embrittlement, making the experimental steel an excellent material for lightweight and high-performance mechanical vehicle applications which could provide a new reference for research on HE susceptibility in ultra-high-strength metallic materials. A further investigation will be carried out to clarify the hydrogen diffusion coefficients and to find the critical hydrogen content of the test steel by charging more hydrogen into the material to reveal the mechanism of its better hydrogen embrittlement resistance.

## 5. Conclusions

(1) The newly developed 30Mn2MoTi ultra-high-strength steel exhibits good strength and plasticity. Its microstructure primarily consists of fine lath martensite with small-size TiC particles dispersed within the matrix, resulting a refined grain size of about 4 μm.
(2) With the extension of charging time, the hydrogen content in the steel increases. The value was 0.35 wppm when the charging time reached 96 h. On the contrary, the fracture mode of the experimental steel remained ductile fracture, and the SSRT strength, the elongation after fracture, and the section shrinkage showed little decrease.
(3) The fine microstructure of 30Mn2MoTi steel contributes to a large number of grain boundaries, which can act as hydrogen traps, promoting the dispersion of hydrogen atoms and reducing the aggregation of hydrogen inside the grains. Furthermore, due to the low content of impurity elements such as S and P, the hydrogen charged into the steel is difficult to interact with, resulting in low hydrogen embrittlement susceptibility.
(4) This manuscript studies the HE susceptibility of the newly developed 30Mn2MoTi ultra-high-strength steel. the results show a positive effect of fine structure, micro-alloy carbides, and cleanliness on resistance to HE, which could provide a new reference for research on HE susceptibility in ultra-high-strength metallic materials. A further investigation will be carried out to clarify the hydrogen diffusion coefficients and to find the critical hydrogen content of the test steel by charging more hydrogen into the material to reveal the mechanism of its better hydrogen embrittlement resistance.

## Figures and Tables

**Table 1:** Chemical composition of experimental steel (wt. %).

Steel	C	Si	Mn	S	P	Ti	Mo	B	Fe
30Mn2MoTi	0.30	0.4	2.0	0.002	0.009	0.06	0.35	0.002	Bal.

**Table 2:** Tensile mechanical properties of experimental steel.

Steel	*Rm* [MPa]	*Rp0.2* [MPa]	*A* [%]	*Z* [%]	*KV_2_* [−40 °C, J]	Yield Ratio
30Mn2MoTi	1769 ± 3	1520 ± 2	12.5 ± 0.5	44.5 ± 1.0	26 ± 1	0.86

**Table 3:** Hydrogen contents and peak temperatures under different charging conditions.

Charging Time [h]	Peak 1 [°C]	Peak 2 [°C]	Peak 1 [wppm]	Peak 2 [wppm]
0	—	370.1	—	0.03
24	142.1	354.0	0.25	0.05
48	144.7	352.9	0.28	0.05
96	145.8	342.0	0.35	0.03

**Table 4:** Corresponding mechanical properties under different charging conditions obtained by SSRT.

Charging Time [h]	Maximal Loading [N]	Displacement at Failure [mm]	*Rm* [MPa]	*A* [%]	*Z* [%]
0	34,600	3.75	1769 ± 3	12.5 ± 0.5	44.5 ± 0.5
24	34,636	3.70	1764 ± 2	12.0 ± 1.0	43 ± 1.0
48	34,714	3.75	1768 ± 4	12.5 ± 0.5	44 ± 0.5
96	34,773	3.55	1771 ± 3	11.5 ± 0.5	42 ± 1.0

**Table 5:** Fracture of steel with different strength after charging.

Steel	*Rm* [MPa] (Uncharged)	Hydrogen Content [wppm]	*Rm* [MPa] (Charged)	*A* [%]	Fracture Mode	Ref.
AISI 4135	1450	0.27	1310	—	brittle fracture	[2]
Low-Ti	2050	0.25	1803	1.9	brittle fracture	[23]
45CrNiMoVA	2160	0.22	1230	0	brittle fracture	[24]
30Mn2 MoTi	1769 ± 3	0.35	1768	11.5	ductile fracture	This article

**Table 6:** Chemical phase analysis results of carbides in the experimental steel (wt.%).

Steel	M_3_C					MC			
	Fe	Mn	Mo	C	Σ	Mo	Ti	C	Σ
30Mn2MoTi	0.426	0.006	0.036	0.032	0.490	0.044	0.060	0.021	0.125

## Data Availability

The original contributions presented in this study are included in the article. Further inquiries can be directed to the corresponding authors.
